# Surface Treatment of Zn-Mn-Mg Alloys by Micro-Arc Oxidation in Silicate-Based Solutions with Different NaF Concentrations

**DOI:** 10.3390/ma14154289

**Published:** 2021-07-31

**Authors:** Shineng Sun, Guo Ye, Ziting Lu, Yuming Weng, Guofeng Ma, Jiatao Liu

**Affiliations:** 1 Institute of Innovative Science and Technology, Shenyang University, Shenyang 110044, China; guo_ye1747111@126.com (G.Y.); luziting1847125@163.com (Z.L.); yumingwen1747119@yeah.net (Y.W.); guofma@163.com (G.M.); 2 School of Materials Science and Engineering, Northeastern University, Shenyang 110819, China; 3 Chinalco Shenyang Non-Ferrous Metals Processing Co., Ltd., Shenyang 110108, China; liujiatao1986@126.com

**Keywords:** zinc manganese magnesium alloys, biodegradable metals, micro arc oxidation, corrosion behavior, NaF concentration

## Abstract

Newly developed Zn-Mn-Mg alloys can be invoked as biomedical materials because of their excellent mechanical properties. However, the corrosion behavior of Zn-Mn-Mg alloys was still lacking in research. It had grown to be a hot research topic to improve the corrosion behavior of Zn alloys by surface treatment to meet the application of degradable Zn alloys in biomedical applications. Micro arc oxidation (MAO) is a simple and effective method to improve the corrosion behavior of the alloy. MAO coatings were successfully prepared on the surface of Zn-Mn-Mg alloys by MAO in silicate-based solutions with different NaF concentrations. The microstructure and phase composition of MAO coatings prepared on Zn-Mn-Mg alloys with different NaF concentrations in the electrolyte was examined by a scanning electron microscope and X-ray diffraction. The results showed that the MAO coatings are porous and mainly composed of ZnO. With the increasing NaF concentration in the electrolyte, the average thickness increases. The distribution of the micro/nanopores was uniform, and the pore size ranged from the submicron scale to several micrometers after MAO treatment in the electrolyte containing different concentrations of NaF. Potential dynamic polarization curves and electrochemical impedance spectroscopy were employed to assess the corrosion behavior of MAO coatings in Hank’s solution. The highest corrosion rate can be achieved after MAO treatment, with an electrolyte concentration of 1.5 g/L NaF in Hank’s solution. These results indicated that MAO coating can accelerate the corrosion resistance of a Zn-Mn-Mg alloy.

## 1. Introduction

Recently, Zinc (Zn) and Zn alloys have been extensively used in biomedical fields, such as cardiovascular stents, bone implants, wound healing equipment, and orthopedic fixation devices [1,2]. This is because of their superior properties of formability and suitable biocompatibility [3,4]. Moreover, Zn can promote bone growth and mineralization, and effectively inhibit the absorption of osteoclasts [5]. However, Zn alloys have several obvious disadvantages [6,7]. For example, considering the recovery process of damaged tissue, the corrosion rate of Zn alloys was relatively slow [8]. So far, Yang has implanted pure zinc stents into the abdominal aorta of Japanese rabbits for a more than perfect degradation period [9]. Therefore, it is urgent to speed up the corrosion rate of Zn alloys.

The common ways to improve the corrosion rate of Zn alloys included alloying and surface treatment [10,11]. Liu reported that the corrosion rate of Zn-Mn alloys was significantly higher than that of Zn-Mg alloys [12]. Recently, researchers found that adding magnesium (Mg) and manganese (Mn) alloy elements to pure zinc greatly improved the mechanical properties of the alloy and also increased the corrosion rate [13,14]. Alloying can accelerate the corrosion rate of Zn alloys, but it is still lower than the ideal corrosion rate (0.1–0.5 mm/a). Therefore, it has grown to be a hot research topic to improve the corrosion behavior of Zn alloys by surface treatment to meet the application of degradable Zn alloys in biomedical applications. There are several ways to form coatings on materials. The methods include techniques such as micro-arc oxidation (MAO), magnetron sputtering deposition, electrophoretic deposition, anodic oxidation, electrolytic polishing, galvanizing process, screen printing, powder coating, water transfer printing, electrophoresis, etc. [15,16,17,18,19,20,21,22,23,24]. Among these methods, MAO is of great interest because of the advantages of a simple process, no pollution, no need for vacuum or low-temperature conditions, etc. But its disadvantage is a high energy consumption [25].

As an effective surface modification method, MAO has been widely used in aluminum (Al), Mg, and titanium (Ti) alloys [26,27,28,29]. The electrolyte composition plays an important role in the process of MAO. The common electrolyte is silicate-based solutions [30,31]. Due to in situ growth on the surface of Mg alloys, the externally porous and internally dense coating structures have been proven to effectively slow the corrosion rate of Mg alloys in the corrosion process [32]. Recently, it has been observed that the corrosion rate of pure zinc can be increased by MAO because of its different surface morphology [33]. However, the corrosion rate was still relatively slow and has yet to be improved. Meanwhile, in order to defeat the shortcoming of high voltage in the MAO process, the sodium fluoride (NaF)-containing silicate-based solutions was developed in the preparation of alloy coating [34,35]. Some researchers have found that the coating thickness increased with an increasing NaF concentration, which significantly reduced energy consumption [36,37]. Therefore, NaF as an additive in silicate-based solutions has been widely studied in the MAO process [38].

In this project, the influence of the NaF concentration in silicate-based solutions on corrosion properties of MAO coating on a Zn-Mn-Mg alloy was investigated. The MAO process was utilized to control the corrosion rate of the Zn-Mn-Mg alloys, so as to meet the application of biodegradable metal materials in biomedical fields. This research can provide technology for the subsequent preparation of biodegradable Zn alloys with an appropriate corrosion rate and a theoretical basis.

## 2. Materials and Methods

### 2.1. Zn-Mn-Mg Samples

Zn-1Mn-0.1Mg alloys were prepared by water-cooled iron mold casting with pure zinc (99.99%), Zn-1Mg (mass fraction) master alloys, and Zn-10Mn (mass fraction) master alloys. The ingots were machined into 20 mm thick plates and then hot-rolled to 1 mm thick sheets with 15% rolling reduction after preheating to 300 °C for 0.5 h. The Zn-Mn-Mg alloy samples were processed into a substrate with dimensions of 20 × 20 × 1 mm^3^. The substrates were ground with SiC sandpapers (Hermes BW114, Guangzhou, China) with 400, 800, 100, 1200, and 1500 granulation, and then polished by a diamond paste of 1.5 μm. The metallographic grinding and polishing machine (UNIPOL-820, Weiyee, Shenyang, China) with a rotation rate of 15 r/s was used in the experiment. All samples were rinsed with distilled water before MAO treatment.

### 2.2. MAO Process

To obtain an MAO coating, the Zn-Mn-Mg alloys were modified by MAO. The MAO process used a pulse power supply (PWR 800H, Kikusui, Japan) with a Zn-Mn-Mg alloy substrate as an anode and a 316L stainless steel disc as a cathode. The basic silicate-based solutions (2000 mL) included 1 g/L KOH and 30 g/L Na_2_SiO_3_ in distilled water. The NaF concentration varied in range from 0.5 g/L to 2.5 g/L. The pulse voltage was 200 V with a treatment time of 5 min. The constant frequency is 1000 Hz and the duty cycle is 20%. The electrolyte temperature was held at 25 °C by adjusting the cooling water flow during the MAO process. After that, the samples were washed with alcohol and dried with an electric hairdryer.

### 2.3. Microstructure and Composition of MAO Coatings

The scanning electron microscope (SEM), with an aS-4800 microscope, operated at a nominal voltage of 15 kV (JEOL, Tokyo, Japan), was applied to observe the surface. The porosity and the diameter of the holes in the MAO coating were analyzed by ImageJ software (ImageJ software, Bethesda, MD, USA). The composition characterization was distinguished by an X-ray diffraction (XRD) diffractometer (Empyrean, PANalytical, Eindhoven, Netherlands) using Cu K_α_ radiation with a scan rate of 3°/min and scanning range of 20°~80°. The diffraction peaks of the MAO coating were analyzed by the Jade 6.0 software (Materials Data, Inc., Santa Clara, CA, USA). The MAO coating thickness was measured by the EPK Mini Test 720 coating thick gauge (EPK, Berlin, Germany). Five points were taken on both sides of the samples, and then the average value was taken.

### 2.4. Electrochemical Properties of MAO Coatings

The electrochemical workstation (Chenhua, Shanghai, China) was used in the electrochemical experiment. The three-electrodes system was used in the electrochemical experiment. The working electrode was a Zn-Mn-Mg alloy sample with an exposed surface area of 1 cm^2^, the reference electrode was a saturated calomel electrode, the auxiliary electrode was a platinum electrode. The corrosion behavior of the MAO coating in Hank’s solution was studied by electrochemical impedance spectroscopy (EIS) and the Tafel curve. The potential scanning rate was at the rate of 10 mV/s, and the constant frequency was set at 1000 Hz. The test solution was Hank’s solution with a pH of 7.2–7.4 at 37 °C.

## 3. Results

### 3.1. Microstructure of MAO Coating

**Figure 1:**
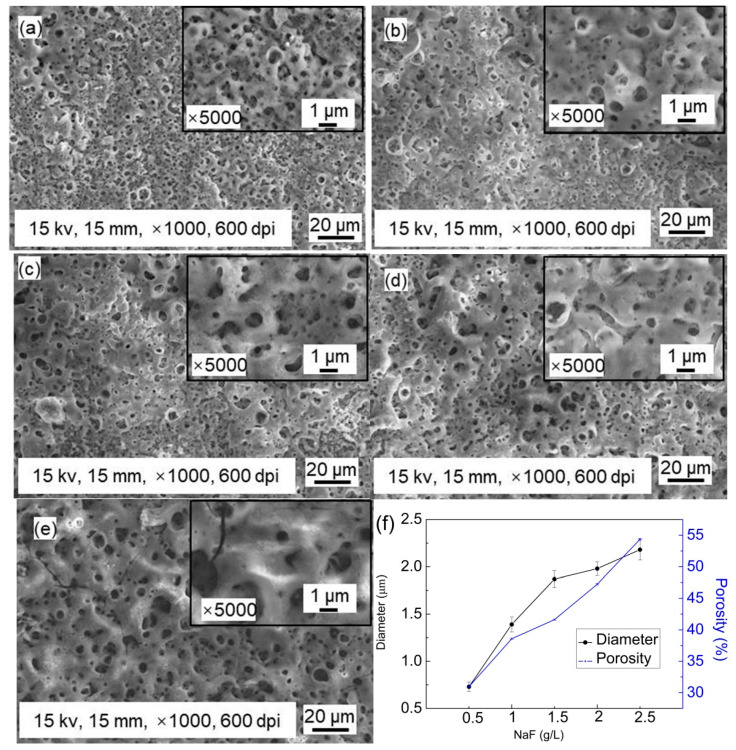
Surface morphologies of MAO coating with different NaF concentrations: (**a**) 0.5 g/L; (**b**) 1 g/L; (**c**) 1.5 g/L; (**d**) 2 g/L; (**e**) 2.5 g/L. (**f**) The curve of the diameter of pores and porosity vs. MAO coating with different NaF concentrations.

Figure 1 shows the surface morphology of the MAO coating prepared on Zn-Mn-Mg alloys with different NaF contents in silicate-based solutions. The surface of all Zn-Mn-Mg alloys after the MAO process presents a porous structure with numerous micropores. The formed coating has the typical surface morphology of MAO coatings [39]. It can be found that the NaF concentration in the silicate-based solutions has a great influence on the formation of the surface morphology of the Zn-Mn-Mg alloys. There are some pores with a diameter ranging from 1.0 to 5.0 µm. With the increasing NaF concentration, the size of porous increases clearly with a decrease in the number of micro-pores. The coating size increases synchronously with the NaF concentration.

The pore diameters of MAO coatings in Figure 1f are measured 50 times by ImageJ software. According to the measurement results, the average value and standard deviation are obtained through statistical analysis. The pores diameters of the MAO coatings are 0.73 ± 0.05 μm (Figure 1a), 1.39 ± 0.08 μm (Figure 1b), 1.87 ± 0.09 μm (Figure 1c), 1.98 ± 0.07 μm (Figure 1d), and 2.18 ± 0.11 μm (Figure 1e), respectively. With an increase in the NaF concentration, the diameter of porous increases, and numerous cracks are formed across the surface. The porosity of the MAO coating is obtained by analyzing the brightness of Figure 1f by ImageJ software. The porosity of the MAO coating is 30.96% (Figure 1a), 38.58% (Figure 1b), 41.61% (Figure 1c), 47.24% (Figure 1d), and 54.35% (Figure 1e), respectively. Therefore, the concentration of NaF in the silicate-based solution plays an important role in the formation of surface morphology.

### 3.2. Thickness of MAO Coating

**Figure 2:**
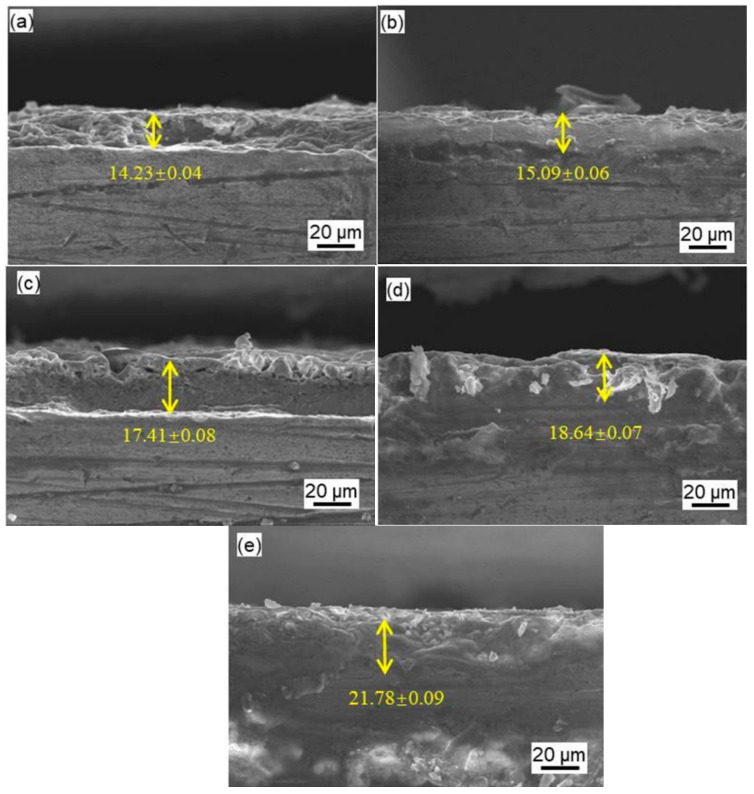
Cross-sectional SEM micrographs of MAO coating with different NaF concentrations: (**a**) 0.5 g/L; (**b**) 1 g/L; (**c**) 1.5 g/L; (**d**) 2 g/L; (**e**) 2.5 g/L.

The coating thickness was measured by a scanning electron microscope. Cross-section microstructures of the MAO coatings are shown in Figure 2. The coating thickness clearly increases when the NaF concentration increases. It is obvious that the average coating thickness augments with NaF contents in the silicate-based solutions from 14.23 ± 0.04 μm after NaF concentrations of 0.5 g/L to 21.78 ± 0.09 μm and after a NaF concentration of 2.5 g/L. Therefore, the NaF concentration has a large effect on the thickness of the MAO coating with the same MAO process.

**Figure 3:**
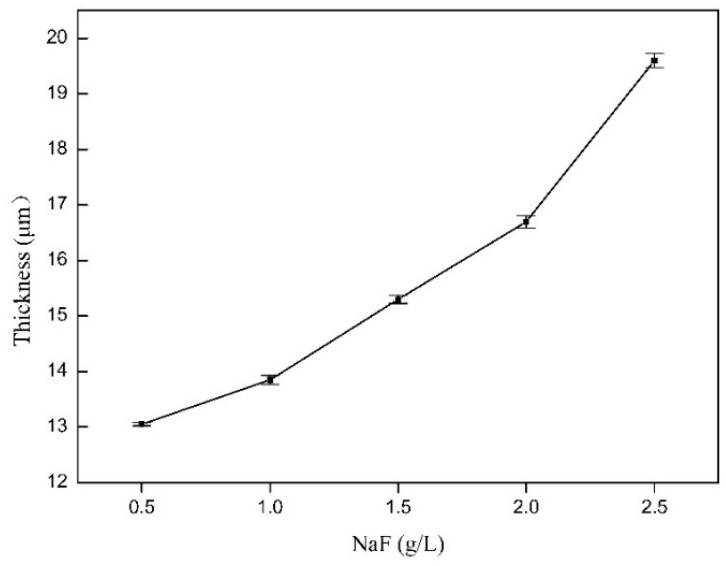
The thickness of the MAO coating with different NaF concentrations from 0.5 g/L to 2.5 g/L.

Figure 3 indicates the thickness of MAO coating prepared on Zn-Mn-Mg alloys with different NaF contents in the silicate-based solutions, as measured by the EPK Mini Test 720 coating thick gauge. The average thickness is 13.05, 13.85, 15.3, 16.7, and 19.6 μm for NaF concentrations of 0.5 g/L, 1 g/L, 1.5 g/L, 2 g/L and 2.5 g/L, respectively. The measured results are in good agreement with the above-obtained data in Figure 2.

### 3.3. XRD of MAO Coating

**Figure 4:**
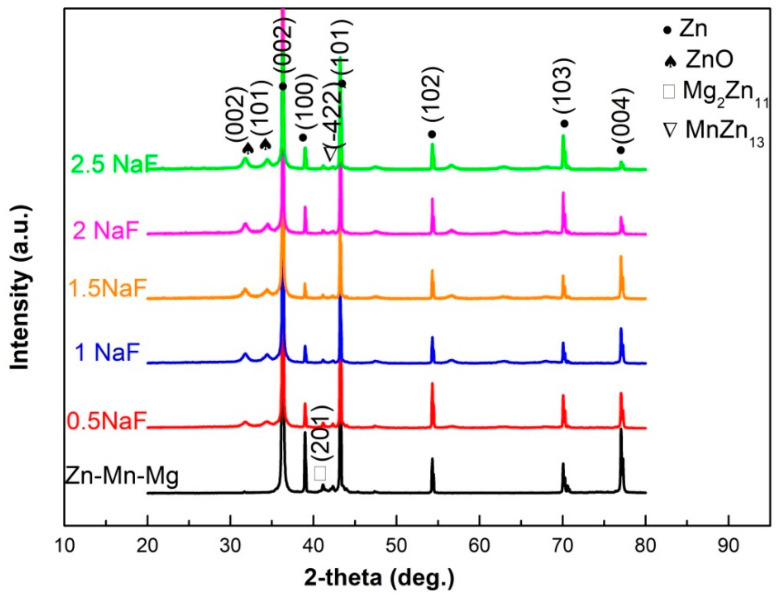
XRD patterns of MAO coating with different NaF concentrations from 0.5 g/L to 2.5 g/L.

In order to investigate the phases of the MAO coating, XRD tests were carried out. XRD patterns of the Zn-Mn-Mg alloy with and without MAO treatment with different NaF contents are shown in Figure 4. The results show that the Zn-Mn-Mg alloys mainly contain Zn, Mg_2_Zn_11_, and MnZn_13_ phases. After the MAO process treatment, the ZnO phase formed. With an increase in the NaF concentration, the intensity of ZnO gradually increases. The formation of the ZnO phase during the MAO treatment process may be determined by the following reaction:

Zn + 2OH^−^ → ZnO + H_2_O + 2e^−^, (1)

### 3.4. Potentiodynamic Polarization Curve of MAO Coating

**Figure 5:**
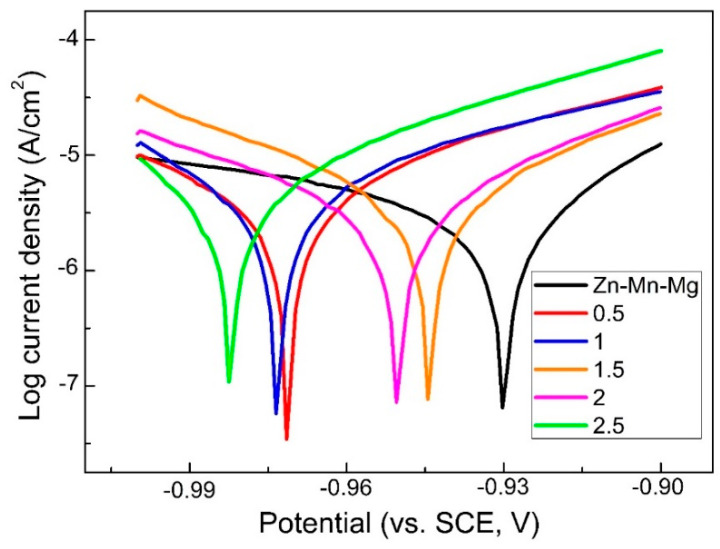
The polarization curves of MAO coating with different NaF concentrations from 0.5 g/L to 2.5 g/L in Hank’s solution at 37 °C.

The polarization curve is widely used to study corrosion behavior because it is suitable for evaluating the corrosion mechanism of MAO coatings [40,41]. Potentiodynamic polarization curves and relevant electrochemical parameters of the Zn-Mn-Mg alloy before and after MAO treatment with different NaF concentrations in the silicate-based solutions in Hank’s solution are exhibited in Figure 5 and Table 1. It can be seen from Figure 5 that hydrogen evolution occurs at the cathode and dissolution of metal/coating occurs at the anode in the polarization curve. Compared with the Zn-Mn-Mg alloys, the corrosion potential of MAO coating samples reduces, indicating that the corrosion resistance of MAO treatment on Zn-Mn-Mg alloys is weakened. According to Table 1, the E_corr_ and i_corr_ were inferred from the polarization curves. For all the polarization curves, the lowest i_corr_ (3.89 μA/cm^2^) can be obtained before MAO treatment. The coating presents a higher i_corr_ than without MAO treatment. The i_corr_ of the MAO coating firstly increases and then decreases with an increase in NaF concentration in the electrolyte. The i_corr_ increases from 5.49 μA/cm^2^ after a NaF concentration of 0.5 g/L to 8.91 μA/cm^2^, after a NaF concentration of 1.5 g/L, and then decreases to 5.42 μA/cm^2^ after a NaF concentration of 2 g/L. The corrosion rates were recorded according to the corrosion current density. The highest corrosion rate (0.133 mm/a) can be obtained after MAO treatment with an electrolyte of 1.5 g/L NaF concentration in Hank’s solution. This indicates that the corrosion rate is significantly improved by the fabrication of coating after the MAO process.

### 3.5. EIS Curve of MAO Coating

**Figure 6:**
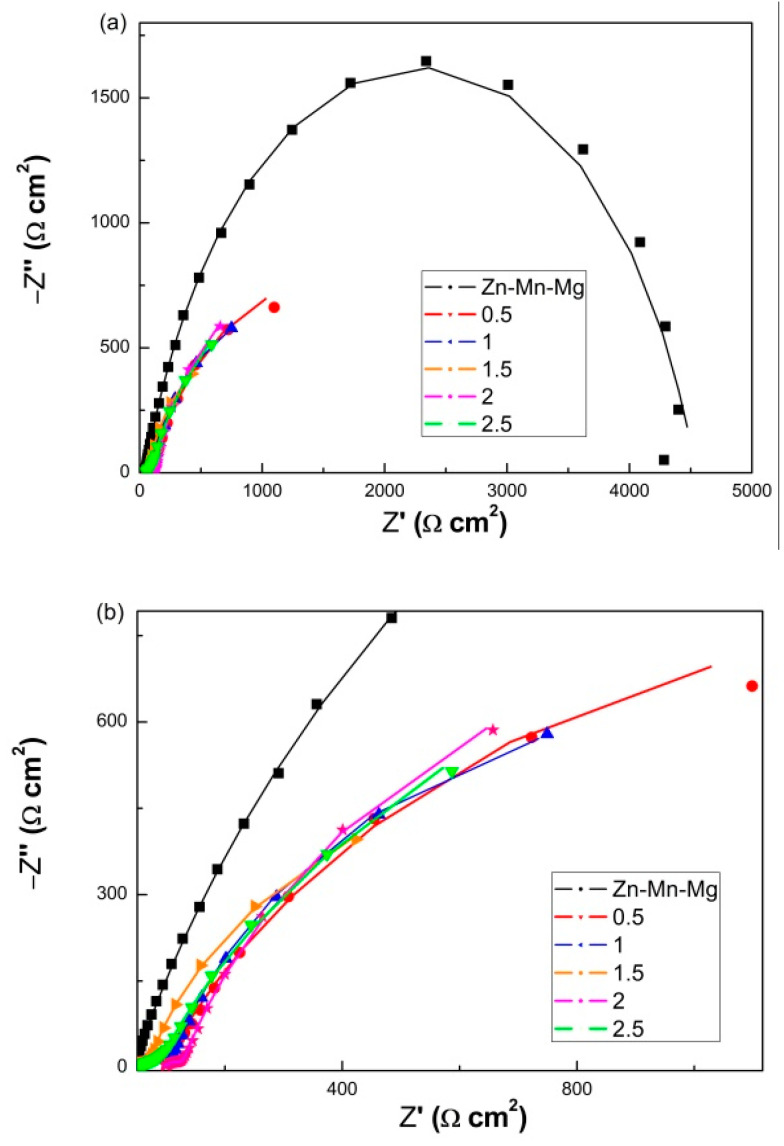
The Nyquist plots of Zn-Mn-Mg alloys and fabricated by the MAO with different NaF concentrations from 0.5 g/L to 2.5 g/L in Hank’s solution at 37 °C: (**a**) Low magnification; (**b**) high magnification.

In order to explore the influencing mechanism of NaF concentrations in the silicate-based solutions on the corrosion behavior of the MAO coating, EIS measurements were employed. EIS plots obtained from the electrochemical test of the Zn-Mn-Mg alloys before and after MAO treatment with different NaF concentrations in Hank’s solution are shown in Figure 6. The EIS curve is a straight line with a slope of 45°, which reflects the Warburg diffusion process. The EIS spectra of Zn-Mn-Mg alloys exhibited capacitive depressed semicircles, which exhibited the biggest impedance loop among the samples. Besides, an inductive loop can be observed at low frequencies [42]. After being modified by MAO, the capacitance circuit decreases, and the impedance value declined markedly. Especially, the corrosion resistance of Zn-Mn-Mg alloys treated with 1.5 g/L NaF concentrations was greatly reduced. This is consistent with the lowest corrosion rate of the polarization curve result (Figure 6b). With an increase in NaF concentrations in the silicate-based solutions, the diameter of the capacitor increases first and then decreases.

**Figure 7:**
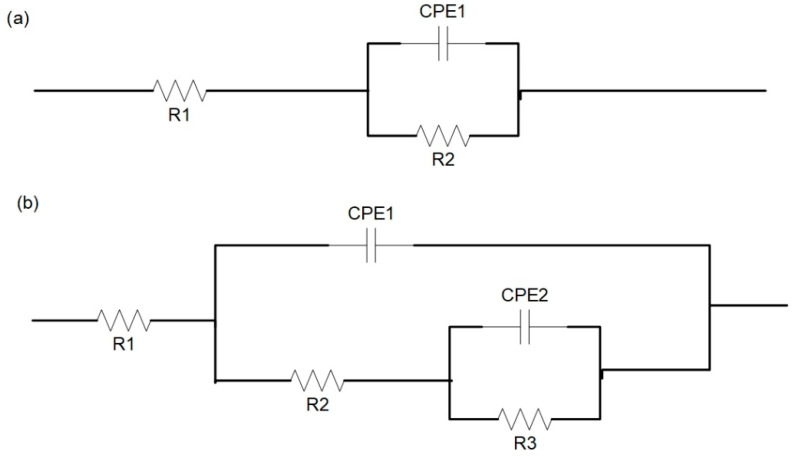
The EIS data equivalent circuit of the Zn-Mn-Mg alloys and fabricated by the MAO with different NaF concentrations from 0.5 g/L to 2.5 g/L in Hank’s solution at 37 °C: (**a**) Zn-Mn-Mg alloys; (**b**) MAO coatings.

Figure 7 shows the equivalent circuits of Zn-Mn-Mg alloys and MAO modified with different NaF concentrations in the silicate-based solutions for the alloys. Capacitance in the equivalent circuit indicates that there is an oxide barrier formed by air on the exposed Zn-Mn-Mg alloys (Figure 7a), whereas the equivalent circuit with two capacitances means a double-layer coating composed of MAO coatings (Figure 7b). R2 and CPE1 represent the resistance and capacitance of the oxide barrier layer, respectively; R3 and CPE2 correspond to the resistance and capacitance of the MAO layer, respectively; R1 is the resistance of the electrolyte between the working electrode and the reference electrode. CPE is calculated by Equation (2), where Z_CPE_ is the CPE impedance (Ω cm^2^), j is the imaginary number, and ω is the angular frequency [43].

CPE = (Z_CPE_)^−1^(jω) ^−n^ (2)

The corresponding parameters are summarized in Table 2.

The total resistance (Rt) of the MAO coating can be calculated using Rt = R2 + R3. A lower Rt value corresponds to worse corrosion resistance. The values are 2985.33, 2309.75, 1893.9, 2618.51, and 3603.28 Ω cm^2^ for the Zn-Mn-Mg alloys with an increase in the NaF concentrations in the electrolyte from 0.5 g/L to 2.5 g/L. Note that the alloys that were MAO treated with 1.5 g/L NaF concentrations, show the minimum Rt, which indicates the worst corrosion resistance of Zn-Mn-Mg alloys. However, it is also observed that the Zn-Mn-Mg alloys before the MAO treatment exhibits maximum resistance (4479 Ω cm^2^). This implies that the MAO treatment can accelerate the corrosion of the Zn-Mn-Mg alloys and that 1.5 g/L NaF is the most effective electrolyte formula.

## 4. Discussion

**Figure 8:**
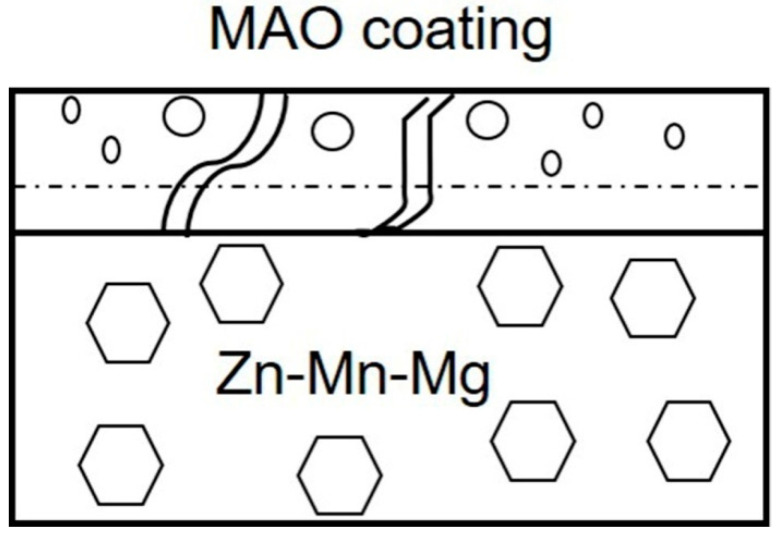
The schematic illustration of Zn-Mn-Mg alloys fabricated by the MAO with different NaF concentrations from 0.5 g/L to 2.5 g/L.

MAO technology can obtain a high degree of combination with alloys, and effectively improve the surface morphologies of Zn-Mn-Mg alloys. A bilayer structure is composed of an outer porous layer and an incoherent dense inner layer, which was formed after MAO treatment on the surface of Zn-Mn-Mg alloys (Figure 8) [33]. According to the oxidation law, the growth process of MAO coating on Zn-Mn-Mg alloys can be classified into different periods. First, a thin oxide coating (barrier layer) is formed on the outside at a low anode voltage without sparking at this stage [44,45]. When the voltage increases and crosses the breakdown voltage threshold, the loss of dielectric stability leads to the formation of discharge passages in the low dielectric region [46]. As a result, many micro sparks are produced in the thinner or weaker speckles and move rapidly to the oxide film [47,48]. Although the period of each spark is very short, the anode film can be formed in the discharge channel and the substrate can be melted [49,50]. When the melt is cooled in the electrolyte, part of the melt spurts along the discharge channel, and the deposit of the molten product on the wall of the discharge channel [51]. In this way, the coating thickness increases, and a porous structure is established. When the voltage exceeds the breakdown potential, an inner layer is formed at the initial stage of the spark [52,53]. The inner layer is loosened with many flaws and holes distributed over the cross surface. Therefore, it is hypothesized that these faults in the anodic coatings occur in low voltage and lead to the inner structure that is formed at the initial spark discharge stage in the case of high gas release and micro-arc discharge. With the formation of a large number of bubbles on the surface of the metal sample, the metallic luster of the substrate gradually disappears, and a very thin oxide coating is formed [54,55].

During the corrosion process, the internal layer is considered as the main protective layer of the MAO coating, which is a continuous protective screen to prevent charge transfer and subsequent degradation [56]. The holes and microcracks produced in the process of MAO provide a channel for the corrosive medium to enter the substrate and form a local galvanic cell. The inner layer can provide a very poor physical isolation of the Zn substrate from the corrosive medium at the beginning of immersion [33]. Due to the mechanical action of oxygen bubbling and micro-arc discharge, the structure of the MAO coating completely peels off, resulting in poor corrosion resistance [57]. Therefore, the accelerated corrosion rate of MAO coating on Zn-Mn-Mg alloys can be expected. The significant increase in the corrosion rate can be attributed to the uniform distribution of pores, the porosity, the diameter of the holes, and the thickness of the MAO coatings.

## 5. Conclusions

In this research, the corrosion resistance of the Zn-Mn-Mg alloy before and after MAO treatment with different NaF concentrations was compared in Hank’s solution at 37 °C by potentiodynamic polarization tests. Additionally, the microstructures of the MAO coating were examined by SEM and XRD. The MAO coating thickness was measured by coating thick gauge and SEM. The main results are as follows.

1. A porous MAO coating composed of the ZnO compound was successfully prepared on the surface of Zn-Mn-Mg alloys. The MAO treatment with 1.5 g/L NaF concentrations in the silicate-based solutions might be advantageous for surface modification in consideration of the size and distribution of the porous structure.
2. With an increase in the NaF concentration in the silicate-based solutions from 0.5 g/L to 2.5 g/L, the average thickness increases from 13.05 μm to 19.6 μm.
3. The double-layer structure with a porous outer layer and a dense inner layer is formed on the surface of Zn-Mn-Mg alloys, which accelerates the corrosion of the Zn-Mn-Mg substrate. The highest corrosion rate can be obtained after MAO treatment with the electrolyte of 1.5 g/L NaF concentrations in Hank’s solution.

## Figures and Tables

**Table 1:** The results of the polarization curves of MAO coating with different NaF concentrations from 0.5 g/L to 2.5 g/L in Hank’s solution at 37 °C.

NaF (g/L)	Corrosion Voltage (V)	Corrosion Current Density (μA/cm^2^)	Corrosion Rate (mm/a)
-	−0.9305	3.89 ± 0.04	0.058 ± 0.003
0.5	−0.9715	5.49 ± 0.08	0.082 ± 0.005
1	−0.9735	6.46 ± 0.09	0.097 ± 0.006
1.5	−0.9445	8.91 ± 0.07	0.133 ± 0.004
2	−0.9505	5.42 ± 0.06	0.081 ± 0.003
2.5	−0.9825	6.92 ± 0.08	0.103 ± 0.005

**Table 2:** The results of the EIS curves of the Zn-Mn-Mg alloys and fabricated by the MAO with different NaF concentrations from 0.5 g/L to 2.5 g/L in Hank’s solution at 37 °C.

NaF (g/L)	R1 (Ω cm^2^)	CPE1 (μF/cm^2^)	R2 (Ω cm^2^)	R3 (Ω cm^2^)	CPE2 (μF/cm^2^)
-	43.56 ± 0.08	0.79554 ± 0.00006	4497 ± 5	-	-
0.5	50.53 ± 0.11	0.49847 ± 0.00008	85.33 ± 0.09	2900 ± 12	0.75837 ± 0.00008
1	46.45 ± 0.09	0.44991 ± 0.00005	85.75 ± 0.11	2224 ± 9	0.84871 ± 0.0001
1.5	43.92 ± 0.07	0.48519 ± 0.00004	34.9 ± 0.3	1859 ± 6	0.84904 ± 0.00009
2	94.93 ± 0.15	0.50795 ± 0.00005	42.51 ± 0.15	2576 ± 13	0.82355 ± 0.00006
2.5	52.28 ± 0.12	0.47861 ± 0.00003	62.28 ± 0.0.27	3541 ± 12	0.78621 ± 0.00007

## Data Availability

Data is contained within the article.
